# Computational Insights into the Molecular Synergy of Paracetamol and Codeine

**DOI:** 10.3390/life16071104

**Published:** 2026-07-02

**Authors:** Manuel-Ovidiu Amzoiu, Georgeta Sofia Popescu, Denisa Constantina Amzoiu, Maria Viorica Ciocîlteu, Gabriela Rau, Costel Valentin Manda, Andrei Gresita, Oana Taisescu

**Affiliations:** 1 Faculty of Pharmacy, University of Medicine and Pharmacy of Craiova, 200638 Craiova, Romania; manuel.amzoiu@umfcv.ro (M.-O.A.); maria.ciocilteu@umfcv.ro (M.V.C.); gabriela.rau@umfcv.ro (G.R.); valentin.manda@umfcv.ro (C.V.M.); 2 Faculty of Food Eng, University of Life Science “King Michael” from Timisoara, 300645 Timisoara, Romania; sofiapopescu@usvt.ro; 3 Department of Physiology, Faculty of Medicine, University of Medicine and Pharmacy of Craiova, 200638 Craiova, Romania; andrei.gresita@umfcv.ro; 4 Department of Anatomy, Faculty of Medicine, University of Medicine and Pharmacy of Craiova, 200638 Craiova, Romania; oana.taisescu@umfcv.ro

**Keywords:** paracetamol, codeine, molecular docking, drug interaction, synergism, COX-1, COX-2, μ-opioid receptor

## Abstract

Combination analgesic therapy is commonly used to improve pain control, yet conventional molecular docking approaches typically evaluate individual ligands and provide limited insight into potential intermolecular associations between co-administered drugs. In this study, paracetamol, codeine, and their proposed 1:1 and 2:1 non-covalent assemblies were investigated using lipophilicity analysis, molecular docking, short molecular dynamics relaxation, electrostatic potential surface mapping, and HOMO–LUMO analysis. Docking simulations were performed against cyclooxygenase-1 (COX-1), cyclooxygenase-2 (COX-2), and the μ-opioid receptor (MOR). The proposed assemblies produced docking scores that differed from those of the individual compounds, with the most pronounced differences observed for the cyclooxygenase targets. The 2:1 assemblies generally exhibited the most favorable docking scores, whereas the predicted interaction profiles at MOR appeared to be more dependent on molecular orientation. Molecular dynamics relaxation and electronic structure analyses further revealed differences in the energetic and electronic characteristics of the investigated configurations. These findings support the theoretical feasibility of distinct interaction patterns among the proposed paracetamol–codeine assemblies within the applied computational framework. However, the reported docking scores represent relative computational values rather than experimentally validated binding affinities, and the short-timescale molecular dynamics simulations provide only preliminary information regarding conformational stability. Furthermore, the existence and biological relevance of the proposed assemblies under physiological conditions remain to be established. This study provides a computational basis for future investigations of intermolecular associations in multicomponent drug systems.

## 1. Introduction

Paracetamol, also known as acetaminophen, is one of the most widely used analgesic and antipyretic agents worldwide [1]. Although it has been in clinical use for over a century, its precise mechanism of action is only partially elucidated and continues to be a subject of scientific investigation [2]. Unlike classical nonsteroidal anti-inflammatory drugs (NSAIDs), paracetamol exhibits minimal peripheral anti-inflammatory activity, which distinguishes its pharmacodynamic profile [2].

Traditionally, the analgesic and antipyretic effects of paracetamol were attributed to the inhibition of cyclooxygenase (COX) enzymes involved in prostaglandin synthesis [2]. The COX pathway converts arachidonic acid into prostaglandin H_2_, a precursor of various prostanoids that mediate pain, fever, and inflammation. Two primary isoforms, COX-1 and COX-2, are well characterized [3]. Paracetamol appears to inhibit prostaglandin synthesis predominantly within the central nervous system (CNS), rather than in peripheral tissues [4]. This central inhibition reduces the production of prostaglandin E_2_ (PGE_2_) in the hypothalamus, thereby lowering the thermoregulatory set point during febrile states and producing antipyretic effects [4,5]. In the spinal cord and brain, decreased prostaglandin production reduces central sensitization of nociceptive pathways, contributing to analgesia [5,6].

Several studies suggest that paracetamol acts as a reducing co-substrate at the peroxidase site of the COX enzyme complex, particularly under conditions of low peroxide tone, such as those found in the CNS [6]. In inflamed peripheral tissues, where peroxide concentrations are elevated, this inhibitory effect is diminished, which may explain the limited anti-inflammatory activity of paracetamol compared to NSAIDs [6]. The absence of significant peripheral COX inhibition explains why paracetamol does not markedly impair platelet function or cause gastrointestinal mucosal injury at therapeutic doses, in contrast to NSAIDs [6]. In addition to COX modulation, its mechanism of action also involves other central pathways, evidence indicating a role for serotonergic descending inhibitory pathways and interaction with the endocannabinoid system via its active metabolite AM404 [6,7].

Numerous studies have highlighted the potential risks associated with paracetamol use, including hepatic, renal, gastrointestinal, and cardiovascular adverse effects, even when administered within the maximum recommended daily dose of 4 g/day [8]. However, hepatotoxicity remains a major risk in overdose, primarily due to the accumulation of the reactive metabolite N-acetyl-p-benzoquinone imine (NAPQI), which depletes glutathione and induces oxidative damage in hepatocytes [6]. Additionally, the optimal therapeutic benefits of paracetamol may require regular dosing three to four times a day, increasing the risk of unintentional overdose if taken improperly [8]. These concerns become more alarming when considering that the majority of individuals who regularly take paracetamol are unaware of its potential risks.

To address these issues, strategies to educate consumers about the benefits, risks, and alternatives to paracetamol are essential to enable its safe and appropriate use [9]. A viable approach is combining paracetamol with aspirin, ibuprofen, or opioids (such as codeine) to achieve effective pain relief while minimizing the total amount of paracetamol required [9].

Codeine is a naturally occurring methylated phenanthrene alkaloid derived from the opium poppy (Papaver somniferum) and is widely used for its analgesic and antitussive properties [10,11]. Pharmacologically, codeine is classified as a weak opioid agonist [10]. Codeine exerts its principal analgesic effects through agonism at μ-opioid receptors (MORs), which are G protein-coupled receptors widely distributed in the central nervous system (CNS). Activation of MORs leads to inhibition of adenylate cyclase activity, decreased intracellular cyclic adenosine monophosphate (cAMP) levels, opening of inwardly rectifying potassium channels, and inhibition of voltage-gated calcium channels. These intracellular events result in neuronal hyperpolarization and reduced neurotransmitter release, particularly of substance P and glutamate, thereby attenuating nociceptive transmission at both spinal and supraspinal levels [12,13]. Through inhibition of nociceptive neurotransmission and modulation of central pain pathways, codeine provides effective mild to moderate analgesia [12,14]. The fixed-dose combination of paracetamol (acetaminophen) and codeine is widely used in the management of mild to moderate pain that is insufficiently controlled by non-opioid analgesics alone [15]. This association is based on the principle of multimodal analgesia, in which two agents with distinct but complementary mechanisms of action are co-administered to enhance analgesic efficacy while potentially allowing lower doses of each component [15]. The paracetamol–codeine combination exemplifies the pharmacodynamic synergy achieved by targeting different sites within the nociceptive pathway [15].

The rationale for combining paracetamol and codeine lies in their complementary sites of action. Paracetamol primarily exerts a central non-opioid analgesic effect, whereas codeine modulates opioid receptors to inhibit pain signaling at both spinal and supraspinal levels. This dual mechanism enhances overall analgesic efficacy compared with either agent administered alone [16]. Clinical studies have demonstrated that the addition of codeine to paracetamol produces superior pain relief in acute nociceptive pain conditions, such as postoperative pain and dental pain, reflecting an additive or mildly synergistic effect [16,17].

Adverse effects reflect contributions from both components. While paracetamol is generally well tolerated at therapeutic doses, hepatotoxicity may occur in overdose due to accumulation of the reactive metabolite N-acetyl-p-benzoquinone imine (NAPQI) [5]. Codeine-related adverse effects include nausea, constipation, sedation, and, at higher doses, respiratory depression and risk of dependence [14]. The combination should therefore be used at the lowest effective dose and for the shortest appropriate duration [16]. Therefore, the paracetamol–codeine combination constitutes a rational multimodal analgesic approach that synergistically integrates central non-opioid and opioid mechanisms, thereby promoting improved and more effective pain management.

Molecular docking is a computational approach used to predict the preferred binding mode and affinity of small molecules toward specific protein targets. The method simulates the interaction between a ligand and a receptor in order to identify the most energetically favorable binding conformation within the active or allosteric site of the protein [18]. By exploring multiple spatial orientations and conformations, docking algorithms estimate how well a ligand fits into the binding pocket and evaluate the strength of the resulting intermolecular interactions. Over the past decades, molecular docking has become an indispensable tool in modern drug discovery. It enables the rapid in silico screening of large numbers of compounds, significantly reducing the cost and time associated with early-stage experimental testing. This approach is particularly valuable for identifying and optimizing molecules that may modulate proteins involved in specific pathological pathways. Several widely used software platforms (such as AutoDock, DOCK, and HEX) implement diverse search algorithms and scoring functions to predict ligand–receptor interactions and estimate binding affinities [19].

Despite its broad applicability, one of the main limitations of molecular docking lies in the accuracy of the scoring functions used to approximate binding energies. The predictive reliability of these functions depends heavily on the quality of the structural models of both the protein and the ligand, as well as on how well the algorithm accounts for molecular flexibility, solvation effects, and entropic contributions. In recent years, advances in machine learning and deep learning have contributed to the development of improved scoring models capable of providing more accurate affinity predictions and better discrimination between active and inactive compounds [19]. Thus, molecular docking represents a powerful and widely adopted strategy in structure-based drug design. While it offers valuable insights into potential binding mechanisms and helps prioritize promising candidates, it remains a predictive tool. Therefore, experimental validation through biochemical and pharmacological assays is essential to confirm the efficacy, selectivity, and safety of proposed drug candidates [20].

## 2. Materials and Methods

### 2.1. HyperChem Software

Chemical modeling of paracetamol and codeine was performed using HyperChem (v. 8.0.8, HyperCube, Gainesville, FL, USA) [21]. The two-dimensional structures of the studied compounds were constructed within the program and subsequently converted into three-dimensional conformations.

Geometry optimization was carried out using two computational approaches: the MM+ molecular mechanics force field and the semi-empirical PM3 method, both available in HyperChem. Energy minimization was performed using the Polak–Ribiere conjugate gradient algorithm, applying an RMS gradient convergence criterion of 0.1 kcal/(Å·mol). From the optimized conformations obtained under both protocols, the lowest-energy (most stable) structures were selected for subsequent analysis. In addition, the theoretical logP values were calculated using the QSAR (Quantitative Structure–Activity Relationship) module implemented in HyperChem [21]. This parameter was used as an indicator of molecular lipophilicity and potential membrane permeability, providing relevant insight into the pharmacokinetic behavior of the investigated compounds.

### 2.2. HEX Software

Molecular docking simulations were performed using HEX software (version 8.0.0) [22] to evaluate binding interactions between the investigated compounds and the receptor active site. Docking was conducted using the “Shape + Electro” correlation mode, which accounts for both steric complementarity and electrostatic interactions.

In the preliminary docking stage, supramolecular drug assemblies were generated by docking paracetamol and codeine against each other. Two reciprocal configurations were evaluated: in the first, paracetamol was treated as the ligand and codeine as the receptor (paracetamol_codeine), while in the second, codeine was treated as the ligand and paracetamol as the receptor (codeine_paracetamol). For each configuration, the top 100 docking poses were generated and ranked according to docking energy. The resulting non-covalent complexes were then exported and used as dual-drug ligands in subsequent docking simulations against COX-1, COX-2, and MOR. During docking preparation, HEX automatically removed crystallographic water molecules and heteroatoms from the protein structures. Each receptor was reoriented relative to the coordinate origin, and docking scores were calculated based on spatial and electrostatic complementarity. The highest-ranking poses were retained, and the most stable conformations were selected for further inspection based on docking energy and structural plausibility. All receptor structures were retrieved from the Protein Data Bank (PDB) [23].

### 2.3. Molecular Dynamics Simulation Protocol

The molecular system was prepared in HyperChem v8.0.0 by manually assigning partial atomic charges: the selected nitrogen atom was set to +1, while the two oxygen atoms were each assigned a charge of −0.5. Energy minimization was performed using molecular mechanics with the AMBER force field, with additional simulation parameters defined in the software settings [21]. The complex was solvated using a rectangular periodic water box, placing the solute at the center and maintaining a defined minimum solute–solvent distance. Overlapping water molecules were automatically removed. Full geometry optimization, including solvent molecules, was carried out. If convergence was not achieved, the maximum optimization cycles were increased up to 800.

Molecular dynamics simulations were then conducted using a simulated annealing protocol, consisting of heating, equilibration, and optional cooling phases. The system was heated from 100 K to 300 K using velocity rescaling (heat time: 0.1 ps, temperature step: 30 K), followed by a constant-temperature run at 300 K for 0.5 ps. During the simulation, stability was monitored using kinetic energy (EKIN), potential energy (EPOT), total energy (ETOT), and temperature (TEMP) [24]. These short MD simulations (picosecond scale) were performed primarily to locally relax the docking-derived conformations in explicit solvent and to verify the relative stability trends suggested by docking.

A limitation of the present study is that the reported docking energies represent relative scoring values generated by the docking algorithm and should not be interpreted as absolute intermolecular interaction or binding energies. The docking results were used primarily to compare the relative interaction patterns and predicted affinities of the investigated supramolecular assemblies. More rigorous quantum chemical approaches, such as density functional theory, would be required to obtain a more accurate description of intermolecular interaction energies and electronic properties.

### 2.4. Electrostatic Potential Surface Analysis

Electrostatic potential surface (ESP) analysis was performed to evaluate the distribution of electronic charge on the molecular surfaces of paracetamol, codeine, and the investigated supramolecular assemblies. Electrostatic potential maps were generated by calculating the interaction energy between the molecular charge distribution and a positive test charge placed at various points surrounding the molecule. The calculated electrostatic potential was then projected onto the molecular surface and visualized using a color scale, where regions of negative potential correspond to electron-rich areas and regions of positive potential correspond to electron-deficient areas.

The resulting ESP maps were analyzed to identify potential hydrogen-bond donor and acceptor regions, characterize charge distribution patterns, and evaluate how supramolecular assembly formation influences the electronic environment of the constituent molecules.

## 3. Results

### 3.1. Lipophilicity Analysis

Hydrophobicity, commonly referred to as lipophilicity, represents a fundamental physicochemical parameter in modern drug design, as it plays a critical role in determining membrane permeability, absorption, distribution, and overall pharmacokinetic behavior of therapeutic agents [25]. Lipophilicity is commonly quantified by the partition coefficient (logP), which reflects the equilibrium distribution of a compound between a nonpolar (lipophilic) phase and a polar (aqueous) phase. In the present study, the lipophilicity of Paracetamol and Codeine was evaluated by calculating their logP values using the HyperChem software package [21]. These calculated values provide insight into the balance between hydrophilic and lipophilic properties of the investigated compounds and serve as an initial indicator of their potential pharmacokinetic behavior. The resulting partition coefficients are presented in Table 1.

### 3.2. Supramolecular Complex Formation and Initial Docking

The initial phase of this study focused on the molecular modeling of Paracetamol and Codeine using the HyperChem software package. Following structural construction and geometry optimization, the two compounds were further analyzed for their ability to form supramolecular assemblies. Molecular docking simulations were performed using HEX 8.0.0 [22], enabling the generation of non-covalent drug–drug complexes. Within this framework, each compound was alternately designated as ligand and receptor, allowing the exploration of two distinct binding orientations. This approach was adopted to evaluate whether the assignment of ligand–receptor roles influences the stability and interaction energy of the resulting supramolecular complex. The comparative docking energies obtained for these configurations are summarized in Table 2.

**Figure 1:**
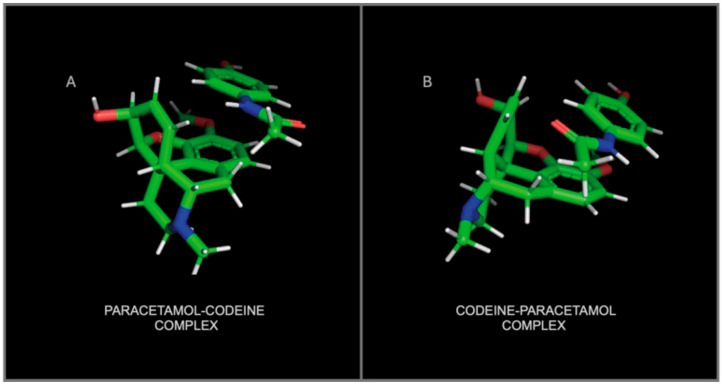
Interaction between paracetamol and codeine visualized with Pymol: (**A**) paracetamol–codeine complex and (**B**) codeine–paracetamol complex.

The results show a slight difference in docking energy between the two orientations, with the codeine–paracetamol configuration exhibiting a marginally lower (more favorable) value, suggesting increased stability relative to the alternative arrangement. Analysis of the binding poses indicates that the two configurations are characterized by distinct interaction patterns. In the paracetamol–codeine orientation, the amide NH group of paracetamol is positioned to act as a hydrogen-bond donor, contributing to stabilizing intermolecular interactions. In contrast, in the codeine–paracetamol configuration, the carbonyl group of paracetamol appears to function predominantly as a hydrogen-bond acceptor, influencing the overall interaction network within the complex [26]. These observations suggest that the differences in docking energy arise from variations in the involvement of key functional groups of paracetamol, particularly the amide and carbonyl moieties, in hydrogen bonding and electrostatic interactions. The results highlight that ligand–receptor assignment can influence the predicted stability and interaction profile of supramolecular drug–drug complexes (Figure 1) [27].

**Figure 2:**
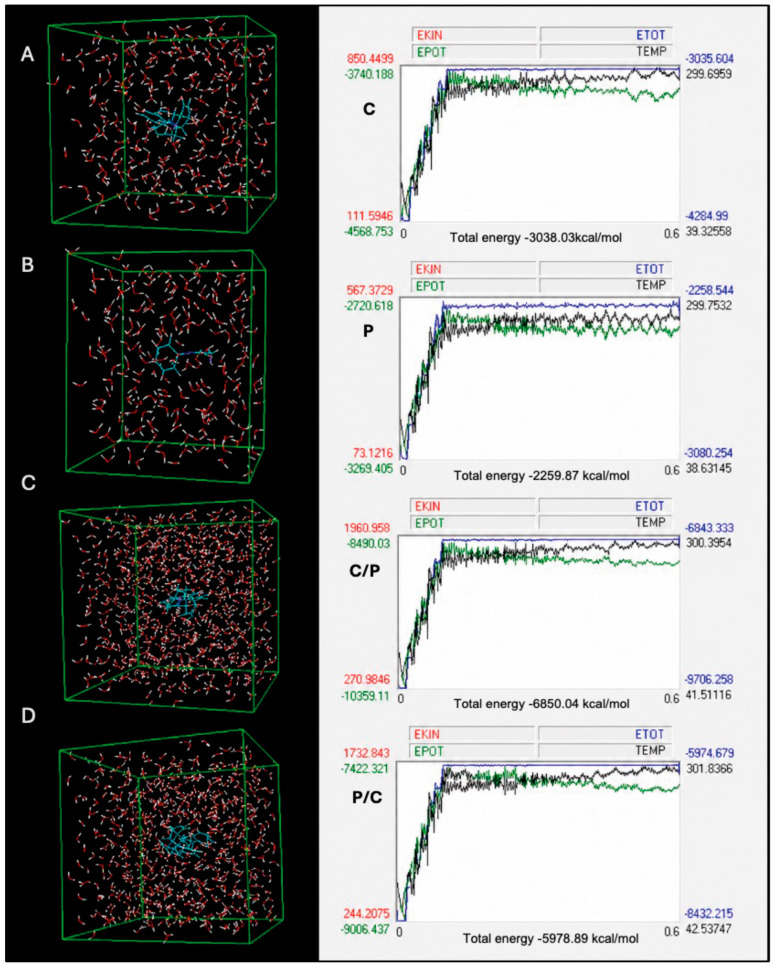
(**A**) Codeine, (**B**) Paracetamol, (**C**) Codeine–Paracetamol supramolecular complex, (**D**) Paracetamol–Codeine supramolecular complex. Total energy values obtained after molecular dynamics simulations for free codeine, free paracetamol, and their supramolecular complexes (1:1) in aqueous environment [21]. The Figure shows the final total energy (kcal/mol) of each compound or complex after MD relaxation, highlighting differences in energetic stability depending on the orientation and composition of the assemblies.

To provide a more comprehensive characterization of the complexation process and to better capture the behavior of supramolecular assemblies relative to the individual compounds, multiple binding variants were generated and evaluated. For the binary systems, formed by the interaction between Paracetamol and Codeine, two docking configurations were considered: paracetamol–codeine and codeine–paracetamol. These orientations were analyzed independently in order to assess whether the assignment of ligand and receptor influences the predicted stability and interaction energy of the resulting complexes (Table 3/Figure 2).

### 3.3. Lipophilicity of Supramolecular Assemblies

To further extend the analysis beyond simple binary interactions, higher-order assemblies were also investigated. In this context, ternary complexes consisting of three molecules were constructed and evaluated. Three distinct structural arrangements were modeled: paracetamol–paracetamol–codeine, paracetamol–codeine–paracetamol, and codeine–paracetamol–paracetamol [28]. These configurations were selected to explore how molecular arrangement, sequence of association, and relative positioning influence the energetic profile, intermolecular interactions, and overall stability of the supramolecular systems.

The partition coefficient (logP) provides important information regarding the balance between hydrophilic and lipophilic properties of a molecule and is directly associated with membrane permeability, solubility, and tissue distribution. In general, compounds with low logP values are more hydrophilic and exhibit higher aqueous solubility but reduced membrane permeability, whereas compounds with higher logP values tend to display enhanced permeability through biological membranes and increased affinity for hydrophobic environments. However, excessively high lipophilicity may negatively affect aqueous solubility and bioavailability. In the present study, paracetamol exhibited a relatively low logP value (0.61), consistent with its known hydrophilic character and rapid systemic distribution, while codeine showed a moderately higher lipophilicity (logP = 1.57), which may contribute to improved membrane penetration and central nervous system accessibility. The increased logP values observed for the binary and ternary supramolecular complexes suggest that intermolecular association enhances the overall hydrophobic character of the systems, potentially influencing their pharmacokinetic behavior, membrane transport, and receptor interaction profiles [21,29].

The results presented in Table 3 provide insight into the lipophilicity and physicochemical characteristics of the investigated compounds. Based on the calculated logP values, both Codeine and Paracetamol exhibit comparable lipophilic profiles in the octanol/water partition system, indicating a similar balance between aqueous solubility and affinity for lipid environments. Notably, when the two compounds are combined into a supramolecular assembly, a slight increase in the partition coefficient is observed. This trend may indicate that the formation of the binary complex leads to a modest enhancement of overall lipophilicity, potentially reflecting cooperative intermolecular interactions between the two molecules. Moreover, the incorporation of an additional paracetamol molecule in the ternary structures results in a further increase in logP, supporting the hypothesis that larger multicomponent assemblies may display greater lipophilic character and improved affinity for hydrophobic environments [29]. These findings may be relevant for optimizing drug formulations intended for pain management, as lipophilicity strongly influences membrane permeability and pharmacokinetic behavior.

Subsequently, the biological targets selected for docking were established. The receptor structures were retrieved from the Protein Data Bank (PDB), using the following entries: 3N8V (COX-1), 5W58 (COX-2), and 6DDF (MOR) [30]. These receptors were chosen due to their central involvement in pain-related pathways. In addition, they represent distinct biological targets with varying binding preferences, allowing evaluation of whether the studied compounds and their complexes exhibit differential affinity toward cyclooxygenase enzymes or the μ-opioid receptor.

Although the calculated logP values indicated similar lipophilicity for both the 1:1 (paracetamol–codeine) and 2:1 (paracetamol-rich) supramolecular complexes, the docking simulations revealed noticeable differences in binding behavior and energetic stability. This apparent contradiction suggests that lipophilicity alone is not sufficient to predict the interaction potential of multicomponent drug assemblies with biological targets.

### 3.4. Molecular Dynamics and Energetic Stability

To better understand the real conformational stability of these complexes and to evaluate how they may behave under conditions closer to the intracellular aqueous environment, we further investigated their properties using molecular dynamics simulations. MD analysis provides additional insight into the energetic relaxation of the systems, the stabilization of intermolecular contacts, and the ability of these supramolecular structures to maintain integrity in solvent conditions that mimic physiological environments.

**Figure 3:**
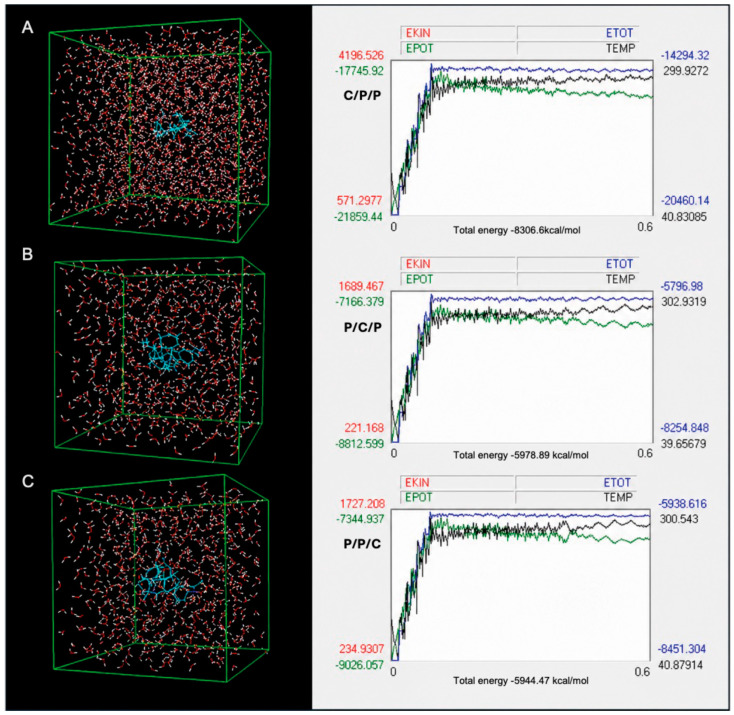
(**A**) Codeine–Paracetamol–Paracetamol supramolecular complex, (**B**) Paracetamol–Codeine–Paracetamol supramolecular complex, (**C**) Paracetamol–Paracetamol–Codeine supramolecular complex. Total energy values obtained after molecular dynamics simulations for free codeine, free paracetamol, and their supramolecular complexes (2:1 stoichiometries) in aqueous environment [21]. The figure shows the final total energy (kcal/mol) of each compound or complex after MD relaxation, highlighting differences in energetic stability depending on the orientation and composition of the assemblies.

The molecular dynamics results summarized in Figure 2 and Figure 3 reveal clear energetic differences between the free compounds and the supramolecular complexes. Individually, codeine (−3038.03 kcal/mol) shows a more negative total energy than paracetamol (−2259.87 kcal/mol), suggesting a slightly higher intrinsic stability of codeine under the applied simulation conditions.

For the 1:1 complexes, both configurations show strongly decreased total energy compared to the isolated molecules, indicating that complex formation is energetically favorable. However, the codeine_paracetamol complex (−6850.04 kcal/mol) is more stable than the paracetamol_codeine complex (−5978.89 kcal/mol). This confirms that the orientation of the molecules within the supramolecular structure significantly influences stability, even though the two complexes have identical composition and comparable lipophilicity.

More importantly, the most striking observation is related to the 2:1 complexes, where the results differ substantially depending on molecular arrangement. Among all tested systems, the codeine_paracetamol_paracetamol complex (−8306.6 kcal/mol) stands out as the most energetically stable structure, displaying a considerably lower total energy compared to the other ternary configurations. This suggests that the presence of two paracetamol molecules may promote a more favorable stabilization network around codeine, likely through enhanced hydrogen bonding, dipole interactions, and cooperative packing effects. In contrast, the other 2:1 complexes show significantly higher energies, such as paracetamol_paracetamol_codeine (−5944.47 kcal/mol), while paracetamol_codeine_paracetamol (−5978.89 kcal/mol) does not show additional stabilization compared to the 1:1 complex. These findings highlight that increasing the stoichiometric ratio does not automatically improve energetic stability unless the structural arrangement allows optimal intermolecular interactions.

It should be noted that the energy values reported in Figure 2 and Figure 3 correspond to the total energy of the simulated systems following molecular dynamics relaxation and do not represent binding or intermolecular interaction energies. These values include the energetic contributions calculated by the selected force field and were used solely for comparative assessment of the relative stability of the investigated assemblies in an aqueous environment.

Overall, these results suggest that although logP values suggest similar hydrophobicity for both 1:1 and 2:1 assemblies, molecular dynamics demonstrates that their stability in aqueous conditions is highly dependent on molecular orientation and interaction geometry. The pronounced stability of the codeine_paracetamol_paracetamol complex supports the hypothesis that specific supramolecular arrangements may form preferentially under physiological-like conditions, potentially influencing their biological performance and receptor binding behavior.

### 3.5. Docking to Pain-Related Targets (1:1 Complexes)

To evaluate whether supramolecular complexation between paracetamol and codeine (1:1 ratio) can influence their biological affinity, molecular docking simulations were performed against three relevant therapeutic targets: COX-1, COX-2, and the μ-opioid receptor (MOR) [31]. These receptors were selected because they represent key molecular pathways involved in pain, corresponding to the pharmacological profiles of the two compounds. Two docking configurations of the 1:1 complex were investigated—codeine_paracetamol and paracetamol_codeine—in order to determine whether the relative orientation of the two molecules within the supramolecular assembly affects binding performance. The docking energies obtained for these complexes were compared with those of free codeine and free paracetamol, allowing assessment of whether complexation improves receptor affinity or alters binding behavior.

The docking results presented in Table 4 indicate that, in most cases, the 1:1 paracetamol–codeine complexes exhibit lower (more favorable) binding energies compared to the individual compounds. This suggests that the supramolecular association between the two molecules may enhance receptor binding through cooperative intermolecular interactions and improved complementarity with the receptor binding pocket.

For COX-1, both complexes demonstrate improved binding affinity compared with free codeine and free paracetamol. The codeine_paracetamol complex (−299.75 kcal/mol) achieved the lowest docking energy, followed by paracetamol_codeine (−283.67 kcal/mol). In comparison, free codeine showed a weaker interaction (−265.81 kcal/mol), while paracetamol alone displayed the least favorable binding (−183.03 kcal/mol) [32]. These findings suggest that the presence of both molecules in a single supramolecular structure promotes more efficient stabilization within the COX-1 active site, likely by increasing the number of contact points and reinforcing non-covalent interactions.

A similar trend was observed for COX-2, where both complexes again exhibited stronger binding than either compound alone. The most favorable interaction was obtained for paracetamol_codeine (−302.33 kcal/mol), slightly outperforming codeine_paracetamol (−296.85 kcal/mol) [32]. This confirms that complex orientation plays a measurable role in binding efficiency, probably due to differences in steric accommodation and exposure of key functional groups to the catalytic pocket.

In the case of MOR, the results show a more nuanced behavior. The codeine_paracetamol complex (−292.71 kcal/mol) produced the strongest docking score, exceeding the affinity of free codeine (−282.82 kcal/mol). However, the paracetamol_codeine complex (−279.41 kcal/mol) displayed a slightly weaker binding energy than codeine alone. This exception is particularly relevant, as it suggests that the presence of paracetamol may interfere with optimal codeine–MOR recognition in one of the configurations. This outcome may be explained by two plausible mechanisms. First, paracetamol could sterically constrain the complex and force codeine to adopt a different orientation, potentially shifting it toward an alternative binding region within the receptor rather than the canonical opioid binding site [33]. Alternatively, even if the complex binds within the same general pocket, the supramolecular arrangement may cause codeine to interact with a different set of amino acid residues, reducing the strength of key stabilizing interactions normally responsible for high-affinity opioid receptor binding. In this configuration, paracetamol may partially block critical contacts, resulting in an overall weaker docking score.

Overall, these results demonstrate that supramolecular complexation generally improves binding affinity toward COX enzymes, supporting the hypothesis of a potential synergistic effect in pain pathways. At the same time, the MOR results highlight that complexation can also modify binding selectivity and interaction geometry, meaning that the paracetamol–codeine association may influence opioid receptor recognition in a configuration-dependent manner.

### 3.6. Docking of 2:1 Complexes

Following the evaluation of the 1:1 supramolecular assemblies, we extended the docking investigation to 2:1 paracetamol–codeine combinations, in order to determine whether increasing the paracetamol proportion further enhances receptor affinity. This step is relevant because paracetamol is often co-administered with opioid analgesics, and a higher paracetamol content could potentially influence the global physicochemical behavior and receptor recognition profile of the resulting supramolecular system. Three different 2:1 configurations were considered: paracetamol_paracetamol_codeine, paracetamol_codeine_paracetamol, and codeine_paracetamol_paracetamol. These were docked against the same therapeutic targets involved in pain modulation, namely COX-1, COX-2, and MOR. The docking energies were compared to those of the individual compounds to evaluate whether complexation improves binding affinity and whether ligand orientation affects receptor interactions.

The results shown in Table 5 demonstrate that all 2:1 paracetamol–codeine complexes exhibit substantially lower docking energies than free codeine and free paracetamol for all investigated receptors. This suggests that increasing the paracetamol content strengthens receptor interactions and improves the overall binding potential of the supramolecular assembly.

For COX-1, the most favorable docking energy was obtained for the paracetamol_paracetamol_codeine complex (−337.71 kcal/mol), followed closely by codeine_paracetamol_paracetamol (−334.28 kcal/mol), and then paracetamol_codeine_paracetamol (−313.91 kcal/mol) [34]. All three complexes bind considerably stronger than free codeine (−265.81 kcal/mol) and paracetamol (−183.03 kcal/mol). This indicates that the presence of two paracetamol molecules may enhance stabilization within the COX-1 active site, likely through additional hydrogen bonding capacity and increased surface complementarity.

A similar pattern is observed for COX-2, where the strongest interaction was again found for paracetamol_paracetamol_codeine (−339.29 kcal/mol). The other configurations also produced highly favorable docking energies: paracetamol_codeine_paracetamol (−332.58 kcal/mol) and codeine_paracetamol_paracetamol (−324.13 kcal/mol). Compared to free codeine (−247.82 kcal/mol) and paracetamol (−186.51 kcal/mol), these results confirm that the 2:1 complexes provide a marked energetic advantage [34]. This supports the hypothesis that supramolecular association increases binding efficiency, potentially by enabling simultaneous interactions of multiple functional groups with residues lining the COX-2 catalytic pocket.

For MOR, all 2:1 complexes also display stronger binding than free codeine. The most favorable docking score was obtained for paracetamol_codeine_paracetamol (−304.94 kcal/mol), followed by paracetamol_paracetamol_codeine (−297.70 kcal/mol) and codeine_paracetamol_paracetamol (−294.96 kcal/mol). These values are consistently lower than codeine alone (−282.82 kcal/mol), suggesting that, unlike the 1:1 case, the 2:1 stoichiometry does not weaken opioid receptor recognition [33]. Instead, the additional paracetamol molecule appears to reinforce the stability of the ligand–receptor complex.

Importantly, these results highlight that the orientation of the three-molecule assembly affects docking performance, but the differences are moderate. The best-performing configuration differs depending on the receptor: COX-1 and COX-2 favor paracetamol_paracetamol_codeine, whereas MOR favors paracetamol_codeine_paracetamol. This suggests that each receptor has distinct steric and electrostatic requirements, and the supramolecular assembly adapts differently depending on how functional groups are exposed to the binding pocket.

Overall, Table 5 confirms that 2:1 supramolecular complexes consistently outperform the individual drugs, indicating a potentially stronger synergistic interaction profile than the 1:1 systems. The increased binding affinity may result from a combination of improved hydrophobic complementarity, additional hydrogen bonding opportunities, and stabilization of the assembly inside the receptor cavity. These findings support the hypothesis that supramolecular association, particularly at higher paracetamol ratios, may enhance binding efficiency toward both COX enzymes and the μ-opioid receptor, potentially contributing to improved pharmacological outcomes in pain management.

### 3.7. Structural Analysis of MOR Binding

To better understand the differences observed in the docking energies of the two 1:1 supramolecular complexes at MOR, a detailed analysis of their receptor–ligand interaction patterns was performed. Although both codeine_paracetamol and paracetamol_codeine contain the same molecular components and identical stoichiometry, their docking scores differed. This suggests that the variation in binding affinity may not arise solely from global physicochemical properties, but rather from differences in how each complex interacts with specific amino acid residues within the receptor binding pocket.

Therefore, the purpose of this structural analysis was to determine whether the two complexes engage distinct sets of amino acids in the MOR binding site and whether these differences could explain the variation in calculated binding energies.

**Figure 4:**
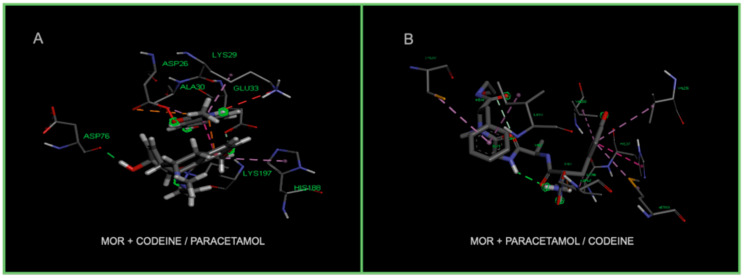
Binding interactions of 1:1 paracetamol–codeine complexes with MOR. (**A**) Interaction pattern of MOR with the codeine_paracetamol complex. (**B**) Interaction pattern of MOR with the paracetamol_codeine complex [35].

Figure 4 illustrates the orientation of the supramolecular complexes within the receptor binding pocket and highlights the amino acid residues involved in stabilizing interactions. The comparative analysis of the two binding models confirms that the differences in docking energies arise from distinct interaction profiles within the MOR binding site. Although both supramolecular assemblies occupy the same general receptor cavity, their internal orientation leads to interaction with different amino acid residues. In the case of the codeine_paracetamol complex (Figure 4A), codeine appears to maintain favorable contacts with key residues typically associated with opioid receptor recognition. These interactions likely include hydrogen bonds, hydrophobic contacts, and possibly π-type interactions that stabilize the ligand within the canonical binding region. The preservation of these critical contacts explains the more favorable docking energy observed for this configuration.

Conversely, the paracetamol_codeine complex (Figure 4B) shows a modified interaction network. The presence and orientation of paracetamol in the leading position appears to alter the spatial arrangement of codeine within the pocket [36]. As a result, codeine interacts with a different subset of amino acids or loses some of the optimal contacts responsible for strong receptor stabilization. In this configuration, paracetamol may partially shield or sterically hinder key interaction sites, forcing codeine to shift slightly within the binding cavity.

Electrostatic potential (ESP) analysis represents an important computational approach for visualizing the electronic distribution on the molecular surface and for evaluating the polarity and lipophilic character of chemical systems. The electrostatic potential may be regarded as an “electronic map” of the molecule, since it reflects the spatial distribution of electron density around the molecular framework. Regions with high electron density correspond to negative electrostatic potential values, whereas electron-deficient regions display positive potential values [37].

Consequently, ESP surfaces provide valuable information regarding possible intermolecular interactions, hydrogen-bond formation, hydrophobicity, and molecular recognition processes. In the present study, electrostatic potential maps were generated for the binary and ternary supramolecular complexes formed between paracetamol and codeine in order to evaluate how molecular arrangement influences the electronic distribution and surface polarity of the resulting assemblies.

**Figure 5:**
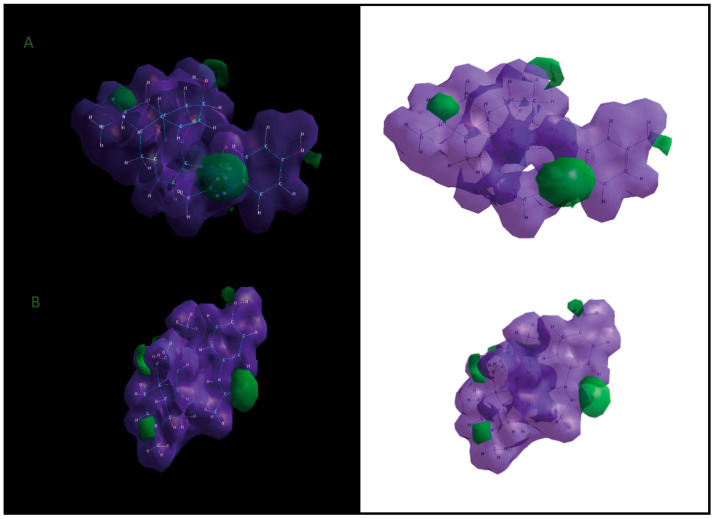
Electrostatic potential maps of the binary paracetamol–codeine supramolecular complexes illustrating the surface distribution of electron-rich and electron-deficient regions that may contribute to intermolecular recognition and receptor interactions [21]. (**A**) Codeine–Paracetamol (**B**) Paracetamol–Codeine.

The electrostatic potential surfaces illustrate the three-dimensional distribution of electron density on the molecular surface. Positive potential regions are represented in green and correspond to electron-deficient areas with increased affinity for polar interactions, whereas negative potential regions are represented in violet and indicate electron-rich, more hydrophobic regions (Figure 5). Slight differences in the spatial distribution of electrostatic potential can be observed between the two complexes, suggesting that the order of molecular association influences the electronic environment and intermolecular interaction profile of the supramolecular systems.

**Figure 6:**
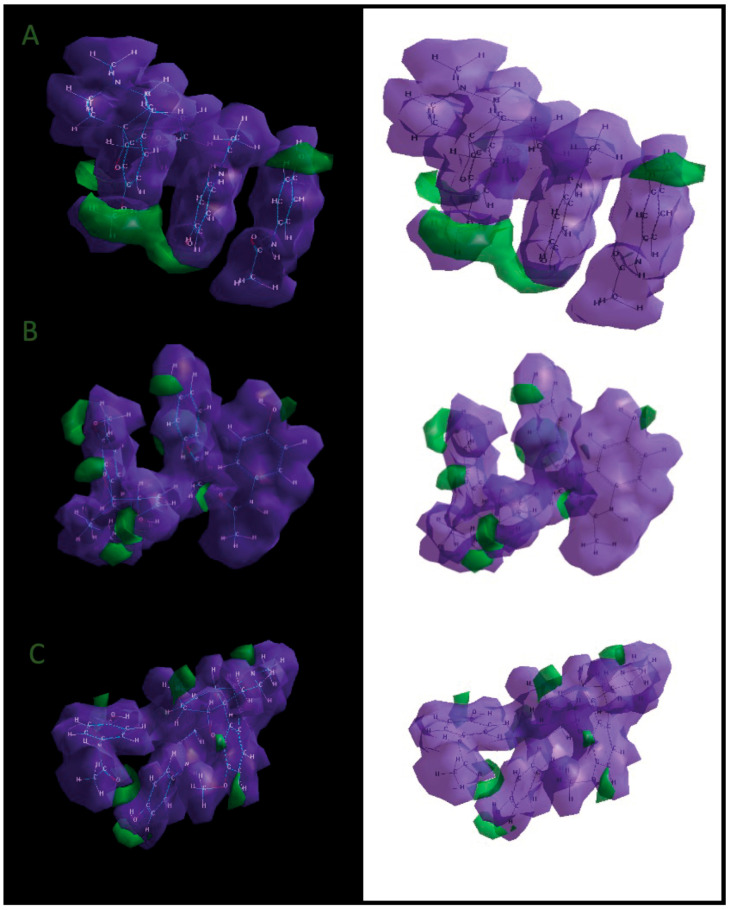
Electrostatic potential maps of the ternary supramolecular complexes: (**A**) Codeine–Paracetamol–Paracetamol, (**B**) Paracetamol–Codeine–Paracetamol, and (**C**) Paracetamol–Paracetamol–Codeine [21].

Positive regions (green) indicate areas capable of favorable polar interactions with the surrounding medium, while negative regions (violet) correspond to hydrophobic electron-rich domains. The observed differences between the three supramolecular assemblies indicate that molecular orientation and sequence significantly influence the global electronic properties, polarity distribution, and potential intermolecular interaction patterns of the complexes (Figure 6).

The electrostatic potential analysis demonstrates that the supramolecular organization of paracetamol and codeine leads to measurable differences in the electronic distribution at the molecular surface. Although the binary complexes contain the same molecular components, the distinct arrangement of the molecules generates slightly different electrostatic profiles. This observation suggests that the order of association influences the local electron density distribution and may consequently affect intermolecular interactions with biological targets or solvent molecules. In particular, the redistribution of positive and negative regions on the van der Waals surface may alter hydrogen-bonding capability, hydrophobic interactions, and molecular recognition processes.

A similar tendency was observed for the ternary complexes, where the addition of a second paracetamol molecule produced more pronounced modifications of the electrostatic surface. The variations in the distribution of hydrophilic and hydrophobic regions indicate that each supramolecular assembly possesses a distinct electronic environment despite its similar chemical composition. These findings support the hypothesis that supramolecular arrangement plays an important role in determining the physicochemical behavior of multicomponent drug systems. Furthermore, the presence of extended hydrophobic regions may contribute to increased membrane affinity, while polar regions may facilitate stabilization through intermolecular hydrogen bonding and interactions with aqueous biological environments.

The frontier molecular orbitals, namely the Highest Occupied Molecular Orbital (HOMO) and the Lowest Unoccupied Molecular Orbital (LUMO), represent essential quantum-chemical descriptors for understanding ligand–receptor interactions and predicting molecular reactivity. Biological interactions between chemical compounds and receptor macromolecules generally involve electron transfer processes occurring in both directions: from the ligand HOMO toward the receptor and from the receptor toward the ligand LUMO. Consequently, the HOMO and LUMO orbitals provide valuable information regarding the electron-donor and electron-acceptor capabilities of bioactive molecules, as well as their chemical stability and reactivity. In the present study, the HOMO–LUMO properties of the binary and ternary supramolecular complexes formed between paracetamol and codeine were investigated in order to evaluate how molecular arrangement influences the electronic behavior and stability of the resulting systems.

The HOMO energy (EHOMO) characterizes the electron-donor capacity and oxidation tendency of the molecule, while the LUMO energy (ELUMO) reflects the electron-acceptor capacity and reduction tendency. Differences observed between the two complexes indicate that the sequence of molecular association influences the electronic distribution and reactivity of the supramolecular assemblies.

**Figure 7:**
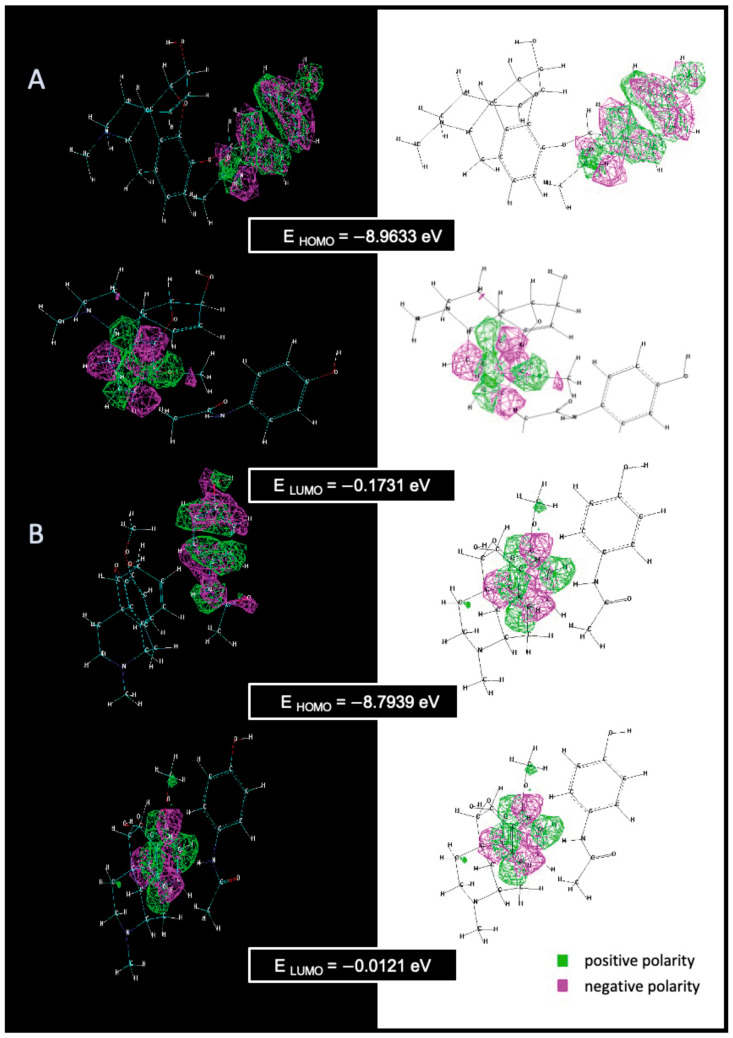
HOMO and LUMO molecular orbitals of the binary supramolecular complexes: (**A**) Codeine–Paracetamol and (**B**) Paracetamol–Codeine.

For the binary complexes presented in Figure 7, the differences indicate that the relative orientation of the molecules within the complex influences the electronic distribution and modifies the electron-transfer potential of the supramolecular assembly. The Paracetamol–Codeine complex, having the higher HOMO energy, may display a greater tendency to donate electrons and participate in electrophilic interactions.

The energy difference between HOMO and LUMO orbitals (ΔE = ELUMO − EHOMO) represents an important descriptor of molecular stability and chemical reactivity. Smaller ΔE values are generally associated with increased molecular reactivity and lower kinetic stability, whereas larger ΔE values indicate more stable systems. Based on the calculated values, the Codeine–Paracetamol complex presents a slightly larger HOMO–LUMO gap compared with the Paracetamol–Codeine arrangement, suggesting a somewhat higher electronic stability.

The calculated HOMO and LUMO energies reveal variations in electronic properties depending on the molecular arrangement within the complexes, suggesting different electron-transfer capabilities and chemical reactivities for each supramolecular system. The HOMO–LUMO analysis revealed noticeable differences in the electronic properties of the investigated supramolecular complexes. The EHOMO descriptor provides information regarding the electron-donor character of the molecule and its susceptibility toward electrophilic attack, whereas ELUMO characterizes the electron-acceptor behavior and susceptibility toward nucleophilic attack. Molecules presenting higher EHOMO values generally exhibit enhanced electron-donating ability, while molecules with higher ELUMO values possess stronger electron-accepting capacity.

**Figure 8:**
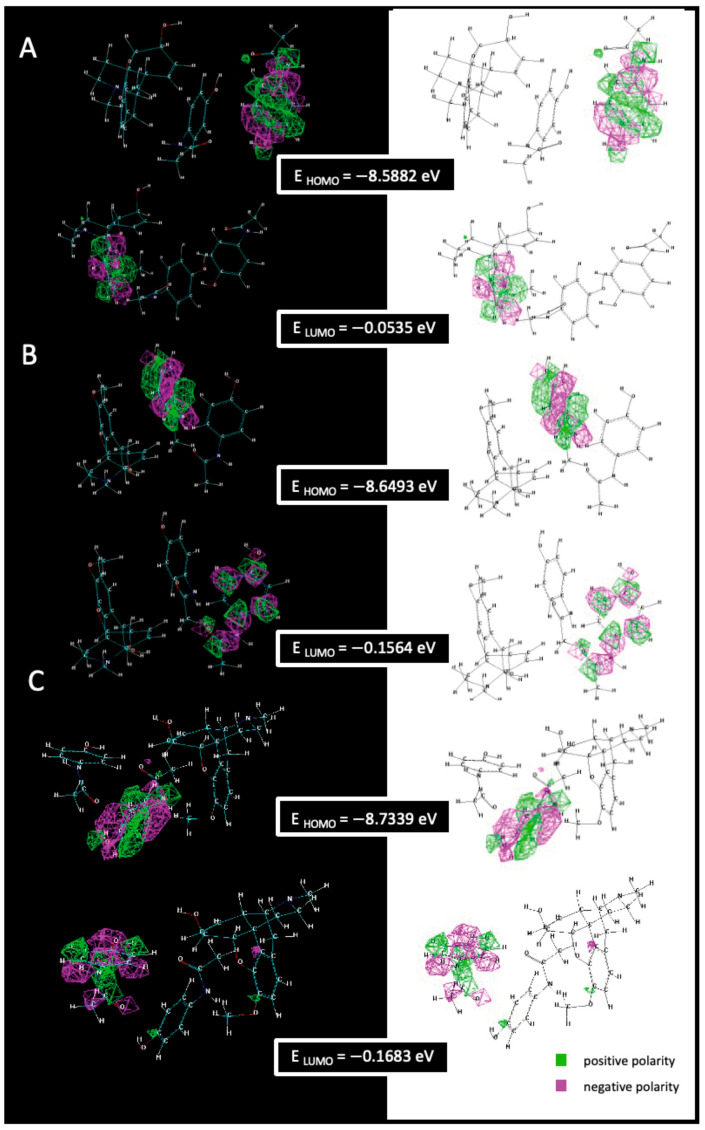
HOMO and LUMO molecular orbitals of the ternary supramolecular complexes: (**A**) Codeine–Paracetamol–Paracetamol, (**B**) Paracetamol–Codeine–Paracetamol, and (**C**) Paracetamol–Paracetamol–Codeine [21].

For the ternary systems shown in Figure 8, additional variations in orbital energies were observed depending on molecular organization. These results indicate that the addition and positioning of a second paracetamol molecule significantly modify the frontier orbital distribution and the electronic properties of the complexes. Among the ternary assemblies, the Codeine–Paracetamol–Paracetamol complex appears to possess the highest electron-donor capability due to its highest EHOMO value. In contrast, the Paracetamol–Paracetamol–Codeine complex, characterized by the most negative LUMO energy, may exhibit enhanced electron-acceptor properties. The differences in HOMO–LUMO gaps between the ternary complexes further suggest that supramolecular arrangement strongly influences molecular stability and reactivity [38]. Overall, these findings support the conclusion that even subtle changes in molecular organization can produce measurable modifications in the electronic behavior of multicomponent drug systems, potentially affecting their interaction with biological receptors and their pharmacological properties.

These structural differences explain why the docking energy for the paracetamol_codeine complex is less favorable compared to codeine_paracetamol, despite their identical composition. The results demonstrate that supramolecular orientation significantly influences receptor recognition, not by changing the overall binding region, but by modifying the specific amino acid interactions that determine stabilization strength. Overall, the figure supports the conclusion that the energetic differences observed at MOR are directly correlated with variations in the amino acid interaction network. Even small conformational or positional changes within supramolecular assemblies can lead to measurable differences in receptor affinity, highlighting the importance of detailed structural analysis when evaluating multicomponent drug systems.

## 4. Discussion

The present study investigated the potential physicochemical and computational interaction profiles of paracetamol, codeine, and their proposed supramolecular assemblies using a combination of lipophilicity analysis, molecular docking, molecular dynamics relaxation, electrostatic potential mapping, and frontier orbital analysis. The objective was to explore whether different molecular arrangements of these compounds could exhibit distinct computational characteristics when evaluated within a consistent in silico framework.

The calculated logP values indicated relatively similar lipophilicity profiles among the investigated supramolecular assemblies. While lipophilicity remains an important physicochemical descriptor related to membrane permeability and distribution, the observed differences in docking scores, total energy values, and electronic properties suggest that lipophilicity alone does not fully characterize the behavior of multicomponent molecular systems. These observations highlight the contribution of molecular organization and intermolecular interactions to the overall computational properties of the investigated assemblies.

The docking analyses revealed differences in the predicted interactions of the binary and ternary assemblies with COX-1, COX-2, and MOR. In general, several supramolecular configurations exhibited more favorable docking scores than the individual compounds within the HEX scoring framework. The most pronounced differences were observed for the cyclooxygenase targets, whereas the MOR results appeared to be more dependent on the spatial arrangement of the molecules within the assembly. Structural analysis suggested that changes in molecular orientation may alter the interaction network established within the receptor cavity, resulting in distinct docking outcomes. However, the reported docking values should be interpreted as relative computational scores rather than experimentally validated binding affinities or interaction energies.

Molecular dynamics relaxation provided additional information regarding the energetic behavior of the investigated systems following short equilibration in an aqueous environment. The observed differences in total energy values indicate that molecular arrangement influences the energetic profile of the assemblies under the applied simulation conditions. Nevertheless, the simulations were performed on a picosecond timescale and were intended primarily for structural relaxation and comparative assessment. Consequently, the results do not permit conclusions regarding long-term conformational stability, persistence in solution, or biological behavior under physiological conditions.

The electrostatic potential and HOMO–LUMO analyses further suggested that different supramolecular organizations produce distinct electronic distributions and frontier orbital characteristics. These findings provide additional information regarding the electronic environment of the assemblies and their potential intermolecular interaction patterns. However, these descriptors should be regarded as complementary computational observations and not as direct evidence of biological activity or receptor modulation.

Taken together, the results suggest that the proposed paracetamol–codeine assemblies exhibit distinct computational characteristics depending on molecular composition and arrangement. The differences observed in docking profiles, energetic parameters, and electronic properties suggest that supramolecular organization may influence the predicted behavior of these systems within the applied computational framework. At the same time, the results remain theoretical and should be interpreted accordingly.

The geometries of the investigated systems were optimized using the MM+ and PM3 methods, which are suitable for rapid conformational screening and comparative analysis but do not provide the level of accuracy achievable with higher-level quantum chemical approaches such as density functional theory. To further characterize the electronic properties of the proposed supramolecular assemblies, electrostatic potential surface analysis was included in the present study. Nevertheless, future investigations employing DFT calculations would provide a more rigorous description of intermolecular interactions and interaction energies.

Another major limitation of the present study is that the proposed paracetamol–codeine supramolecular assemblies are supported exclusively by computational evidence. Although molecular docking, molecular dynamics, HOMO–LUMO, and electrostatic potential analyses indicate the theoretical feasibility of intermolecular association, the existence, stability, and biological relevance of such complexes under physiological conditions have not been experimentally established. Accordingly, the observed results should be regarded as computationally derived interaction models rather than evidence of supramolecular complex formation or pharmacological synergy. Experimental studies employing techniques such as NMR, IR, UV–Vis spectroscopy, DOSY, DLS, and mass spectrometry will be required to evaluate these predictions.

An additional limitation is that the computational approach does not determine whether the proposed supramolecular assemblies remain intact during diffusion in aqueous media or throughout the receptor-binding process. While the investigated configurations were energetically feasible within the applied simulations, their kinetic stability and persistence under physiological conditions remain uncertain. This consideration is particularly relevant for receptors with relatively restricted binding cavities, such as MOR, where partial or complete dissociation of the assemblies prior to receptor recognition cannot be excluded. Consequently, the docking poses presented in this study should be interpreted as theoretical interaction models that facilitate exploration of potential binding scenarios. Further investigation using longer-timescale molecular dynamics simulations, free-energy calculations, and experimental approaches capable of detecting intermolecular association in solution will be necessary to assess the biological relevance of these assemblies.

## 5. Conclusions

The present computational study explored the potential formation and behavior of paracetamol–codeine supramolecular assemblies using molecular modeling, docking, molecular dynamics relaxation, electrostatic potential analysis, and frontier orbital calculations. The results suggest that different molecular arrangements may exhibit distinct physicochemical properties, docking scores, and energetic profiles when evaluated against pain-related targets, including COX-1, COX-2, and the μ-opioid receptor. The docking analyses indicated that certain supramolecular configurations displayed more favorable predicted receptor interactions than the individual compounds within the applied computational framework. In addition, molecular dynamics relaxation and electronic structure analyses suggested that molecular organization may influence the relative stability and interaction patterns of the investigated assemblies. However, the findings should be interpreted with caution. The reported docking scores represent relative computational predictions rather than experimentally validated binding affinities, and the short-timescale molecular dynamics simulations provide only preliminary information regarding conformational stability. Furthermore, the existence and persistence of paracetamol–codeine supramolecular assemblies under physiological conditions have not been experimentally demonstrated. Overall, this study provides a theoretical framework for exploring possible intermolecular interactions between paracetamol and codeine and their potential influence on receptor recognition. Further investigations employing higher-level computational methods, longer molecular dynamics simulations, and experimental validation will be required to determine the biological relevance of the proposed assemblies.

## Figures and Tables

**Table 1:** Chemical structures and calculated octanol/water partition coefficients (logP) of paracetamol and codeine obtained using the HyperChem QSAR module. The calculated values provide an estimate of the relative lipophilicity of the compounds and their potential membrane permeability.

Structure	Compound	logP (Octanol/Water)
Paracetamol	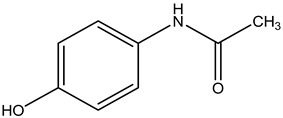	0.61
Codeine	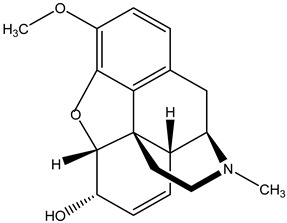	1.57

**Table 2:** Docking energies obtained for binary paracetamol–codeine assemblies generated by reciprocal docking. The two configurations represent alternative molecular orientations resulting from the assignment of each compound as ligand or receptor during supramolecular complex formation.

Receptor	Ligand	Energy (kcal/mol)
Paracetamol	Codeine	−123.30
Codeine	Paracetamol	−123.22

**Table 3:** Calculated octanol/water partition coefficients (logP) of the binary and ternary paracetamol–codeine supramolecular assemblies.

Compound	logP (Octanol/Water)
Codeine–Paracetamol	2.18
Paracetamol–Codeine	2.18
Codeine–Paracetamol–Paracetamol	2.79
Paracetamol–Codeine-Paracetamol	2.79
Paracetamol–Paracetamol–Codeine	2.79

**Table 4:** Docking scores of the 1:1 paracetamol–codeine supramolecular complexes and individual compounds against COX-1, COX-2, and μ-opioid receptor (MOR). Lower docking scores indicate more favorable predicted receptor interactions within the HEX scoring framework [22].

Receptor	Ligand	Energy (kcal/mol)
COX1	codeine_paracetamol	−299.75
	paracetamol_codeine	−283.67
	codeine	−265.81
	paracetamol	−183.03
COX2	paracetamol_codeine	−302.33
	codeine_paracetamol	−296.85
	codeine	−247.82
	paracetamol	−186.51
MOR	codeine_paracetamol	−292.71
	codeine	−282.82
	paracetamol_codeine	−279.41
	paracetamol	−208.92

**Table 5:** Docking scores of the 2:1 paracetamol–codeine supramolecular complexes and individual compounds against COX-1, COX-2, and μ-opioid receptor (MOR), highlighting the influence of molecular arrangement and stoichiometry on predicted receptor binding [24].

Receptor	Ligand	Energy (kcal/mol)
COX1	paracetamol_paracetamol_codeine	−337.71
	codeine_paracetamol_paracetamol	−334.28
	paracetamol_codeine_paracetamol	−313.91
	codeine	−265.81
	paracetamol	−183.03
COX2	paracetamol_paracetamol_codeine	−339.29
	paracetamol_codeine_paracetamol	−332.58
	codeine_paracetamol_paracetamol	−324.13
	codeine	−247.82
	paracetamol	−186.51
MOR	paracetamol_codeine_paracetamol	−304.94
	paracetamol_paracetamol_codeine	−297.7
	codeine_paracetamol_paracetamol	−294.96
	codeine	−282.82
	paracetamol	−208.92

## Data Availability

The original contributions presented in this study are included in the article. Further inquiries can be directed to the corresponding author.
